# Bismuth oxychloride anchored on waste stainless-steel slag: preliminary assessment of its potential as an active photocatalytic support

**DOI:** 10.1007/s11356-025-36656-1

**Published:** 2025-06-24

**Authors:** Siaw Foon Lee, Eva Jimenez-Relinque, Andrea Martinez-Topete, Jorge Ruiz-Fernandez, Marta Castellote

**Affiliations:** 1 Institute of Construction Science Eduardo Torroja, IETcc-CSIC, Madrid, Spain; 2 Escuela Técnica Superior de Ingenieros Industriales, Universidad Politécnica de Madrid (UPM), Madrid, Spain

**Keywords:** Photocatalysis, Bismuth oxychloride, Stainless-steel slag, NOx abatement, Mechanochemical synthesis

## Abstract

Environmental pollution remains a critical barrier to sustainable societal development. In this context, the present work explores a sustainable approach to environmental remediation through the mechanochemical synthesis of bismuth oxychloride (BiOClm) immobilized on waste stainless-steel slags (SSS) at varying proportions. This strategy exemplifies the principles of circular economy by transforming industrial waste into a high-value, photocatalytically functionalized material. The composites were thoroughly characterized in terms of their physicochemical and optical properties, and their photocatalytic activity was evaluated through nitrogen oxide (NOx) abatement under UV and visible light. Although the absolute photocatalytic activity of the composites was lower than that of pure BiOClm, their efficiency per unit of BiOClm was higher, indicating improved utilization of the active phase. This enhancement is attributed to a slight semiconductor behavior of SSS metallic constituents, as confirmed by photocurrent measurements. The coupling between BiOCl and the metal-rich support results in an upward shift of the band energy positions, thereby encompassing the energy of oxygen conversion to superoxide. Thus, the superior photocatalytic activity (normalizes to the photocatalyst mass) of BiOCl_m_ supported on SSS is attributed to the BiOCl–metal–support interactions, which facilitate charge separation and enhance redox performance. The results indicate that BiOCl/SSS composites with BiOCl contents between 20 and 40% exhibit the most effective photocatalytic performance, achieving a maximum NOx removal efficiency of 1.49% per gram, compared to 0.8% for pure BiOCl. According to the results, a proportion of SSS in the range 60–80% would be the optimum to be used as an active support for BiOCl_m_. Overall, this work offers a promising route for the valorization of industrial waste into functional materials for air pollution control.

**Supplementary Information:**

The online version contains supplementary material available at 10.1007/s11356-025-36656-1.

## Introduction

Photocatalytic air purification has been extensively studied over the last 50 years. During this time, several pollutant oxidation processes (Sharma et al. 2022) such as the oxidation of nitrogen oxides (NOx) (Bloh et al. 2014) and volatile organic compounds (VOCs) (Almaie et al. 2022) have been proposed.

Titanium dioxide (TiO_2_) is the archetypal photocatalyst, as it is considered inexpensive, photo-stable, nontoxic, available, and easily activated by near-UV radiation. However, over the last few decades, studies have been focusing on developing novel photocatalysts with improved catalytic properties, enhanced stability, and increased surface area. Several studies have investigated the applicability of different metal semiconductors such as zinc oxide (Abdullah et al. 2022) and iron oxide (Wu et al. 2015), among others.

Bismuth oxyhalides, BiOX (*X* = Cl, Br and I), a new class of promising layered materials for environmental remediation, have been intensively investigated (Di et al. 2017). Compared to traditional metal oxides, they have some advantages such as chemical stability, unique layered structure, and easy preparation (Jimenez-Relinque et al. 2024; Martinez-Topete et al. 2025). In addition, the tendency to form micrometric size structures assembled by large amounts of nanosheets can reduce the possible environmental risk associated with the use of nanoparticle formulations (Nava-Núñez et al. 2022).

Due to the lack of proper photocatalyst disposal strategies and toxicity associated with nanoparticles, the concept of immobilizing photocatalysts on a suitable substrate has proposed as a partial solution (Joseph and Vijayanandan 2023). Recent studies in the field of heterogenous catalysis have focused on identifying suitable anchoring supports for immobilizing commonly employed photocatalysts, mainly TiO_2_ (Joseph and Vijayanandan 2023; Srikanth et al. 2017). Special attention has been given to support materials derived from various types of waste, due not only to their alignment with circular economy principles but also to their environmental benefits in the context of sustainable development. Several waste materials have been explored as supports for immobilizing photocatalysts, including polystyrene particles (Altın and Sökmen 2014; Singh et al. 2015), coal fly ash (Bansal and Verma 2017; Lu et al. 2013), photovoltaic silicon wafers (Teng et al. 2024), and iron tailings (Sun et al. 2023), among others.

Focusing on the use of steel slag, a relevant study described the synthesis of a composite photocatalyst consisting of TiO_2_ supported on slag-derived calcium silicate produced for degradation of dye pollutants in water via a simple mixing process using a blast furnace slag (Shi et al. 2017). More recently, Malekhosseinia et al. demonstrated the photocatalytic degradation of ethylbenzene in water with 99.5% efficiency using ZnFe_2_O_4_ nanoparticles supported on copper slag, designed through co-precipitation and thermal treatment methods (Malekhossini and Mahanpoor 2021). Additionally, another study reported the use of layered double hydroxides (LDHs) based on zinc, aluminum, and either titanium or iron, synthesized using aluminum recovered from industrial saline slag waste, as catalysts for the photodegradation of emerging contaminants such as diclofenac and salicylic acid under UV light (Santamaría et al. 2020). A previous study reported its proper modification with nanostructured palladium on the surface, enabling the conversion of CO_2_ into methanol and hydrogen (Fusco et al. 2022). Notably, this work provides the first evidence in the literature that steel slag can be used as a photocatalyst for CO_2_ photoreduction. However, most of the existing literature has focused on nanoparticle-based photocatalysts for water decontamination, while the potential of these slag-derived materials for air purification remains largely unexplored.

With this motivation, we intend to provide a practical demonstration of the circular economy concept by using an industrial by-product from steel manufacturing: the design of a photocatalytically functionalized material through the anchoring of a BiOCl pure photocatalyst onto waste stainless-steel slag. Stainless-steel slags are mostly composed of CaO, MgO, Al_2_O_3_, and SiO_2_. Previously, we demonstrated the synergetic adsorption–photocatalysis process for water treatment using TiO_2_ supported on stainless-steel slag. Through an acid–base treatment of the slag, we obtained an effective adsorbent for removing methylene blue from wastewater (Plaza et al. 2021). This is, to the best of our knowledge, the first study to explore the use of stainless-steel slag as a support for BiOCl in gas-phase photocatalytic air purification applications. Unlike previous studies that focused primarily on water remediation, our approach targets NOx removal under both UV and visible light, demonstrating significant photocatalytic activity and nitrate selectivity.

## Materials and experimental methods

### Sample preparation

**Fig. 1:**
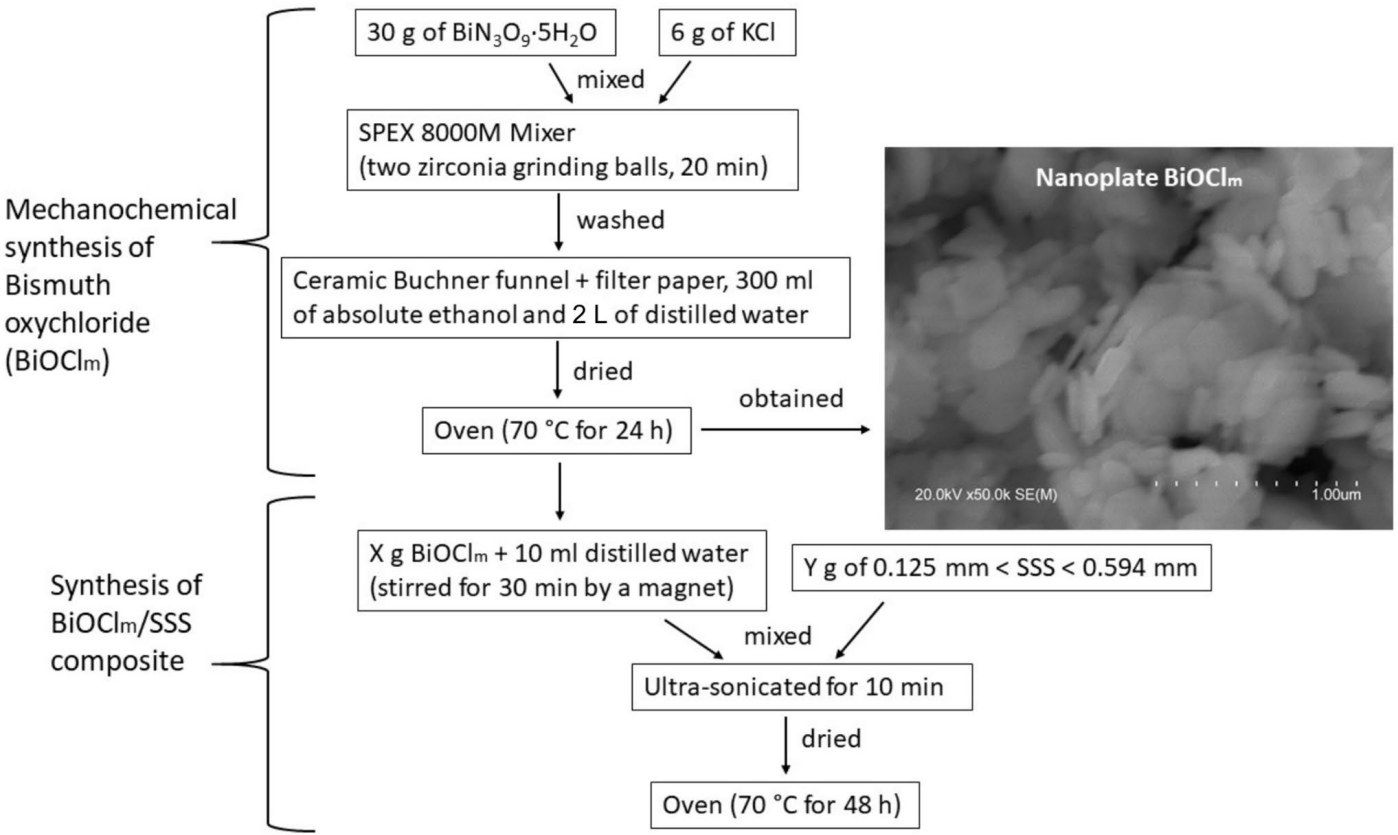
Flow chart of the mechanochemical synthesis of BiOCl_m_ and the synthesis of BiOCl_m_/SSS

Figure 1 shows the flow chart of the mechanochemical synthesis of bismuth oxychloride (BiOCl_m_) and the synthesis of the BiOCl_m_/SSS composite. First, 30 g of bismuth (III) nitrate pentahydrate (Bi (NO₃)₃·5H_2_O) and 6 g of potassium chloride (KCl) were placed in a vial with two zirconia grinding balls. The mixture was then ground for 20 min using a SPEX 8000 M mixer. The resulting white slurry was filtered and washed under pressure with 300 mL of absolute ethanol followed by 2 L of distilled water. The recovery solid was dried at 70 °C for 24 h, yielding nanoplate-shaped BiOCl_m_ (Fig. 1).

The raw SSS was sieved to a size between 0.125 and 0.594 mm. To prepare the composites, *X* grams of BiOCl_m_ powder was dispersed in 10 mL of distilled water and stirred for 10 min to ensure uniform distribution. *Y* grams of sieved SSS was then added into the dispersion. and the mixture was ultrasonicated for 10 min. The resulting mixture was dried at 70 °C for 48 h. The obtained composite powder obtained was designated as *X*gBiOCl_m_ + *Y*gSSS, according to the corresponding mass proportions. A total of six powder samples were fabricated: BiOCl_m_, 8gBiOCl_m_ + 2gSSS, 6gBiOCl_m_ + 4gSSS, 4gBiOCl_m_ + 6gSSS, 2gBiOCl_m_ + 8gSSS, and SSS.

### Sample characterization

Thermogravimetric analysis (TA Instruments: SDT Q600) was performed on the BiOCl_m_ and as-received SSS in the range of 0–1000 °C. All the prepared samples were characterized in terms of the Brunauer–Emmett–Teller–specific surface area, pore structure, and pore size distribution using N_2_ adsorption–desorption isotherm by an ASAP2010 analyzer. X-ray diffraction patterns were obtained using a X-ray diffractometer (D8 Advance Bruker) with copper as the X-ray source, operating at 40 kV and 30 mA. Surface morphology and the elemental mapping of the BiOCl_m_/SSS composite were obtained using a scanning electron microscope with an energy-dispersive X-ray spectroscopy (SEM–EDX: HITACHI S-4800). Photoluminescence spectra were measured at room temperature using a Perkin Elmer LS 55 Fluorescence Spectrometer with an excitation wavelength of 315 nm. Fourier transform infrared spectroscopy (FTIR) was carried out using a Thermo Scientific Nicolet 6700 Spectrometer at room temperature within the 400 to 4000 cm^−1^ range to identify surface functional groups. The band gap energy was determined using UV–Vis diffuse reflectance spectroscopy (UV-2600 spectrophotometer, Shimadzu).

### Electrochemical characterization

Two grams of the sample powder was magnetically stirred with 25 mg of hydroxyethyl-cellulose and 1.5 mL of distilled water for 30 min to form a homogeneous paste. This paste was then used to coat a 1 cm^2^ area on a fluorine-doped tin oxide (FTO) glass slide using the doctor-blade technique. Mott-Schottky analysis was carried out in a three-electrode configuration using a potentiostat/galvanostate Autolab (PGSTAT204) with a Nova 1.10 software. The scan was performed from 0.4 to − 0.8 V, avoiding further cathodic polarization to prevent FTO corrosion. The sample-coated FTO served as the working electrode, a Pt wire as the counter electrode, and an Ag/AgCl (3 M KCl) electrode as the reference. The electrolyte used was 0.2 M Na_2_SO_4_ (pH 6.4) at room temperature.

Photocurrent density–time (I–t) measurements were performed using a two-electrode setup under an applied potential of 130 mV (Y. Long et al. 2018), with the sample-coated FTO as the working electrode and a Pt wire as the counter electrode. Two illumination sources, UV (Philips TL-D 15 W BLB) or visible light (Philips Master, TL-D 18W/840), were used. The photocurrent density was measured under 20-s on–off cycles using the Autolab PGSTAT204 through the Chrono Amperometry (Δ*t* > 1 ms) method in Nova 1.10 software with a data interval of 0.05 s.

### Photocatalytic NOx removal

**Fig. 2:**
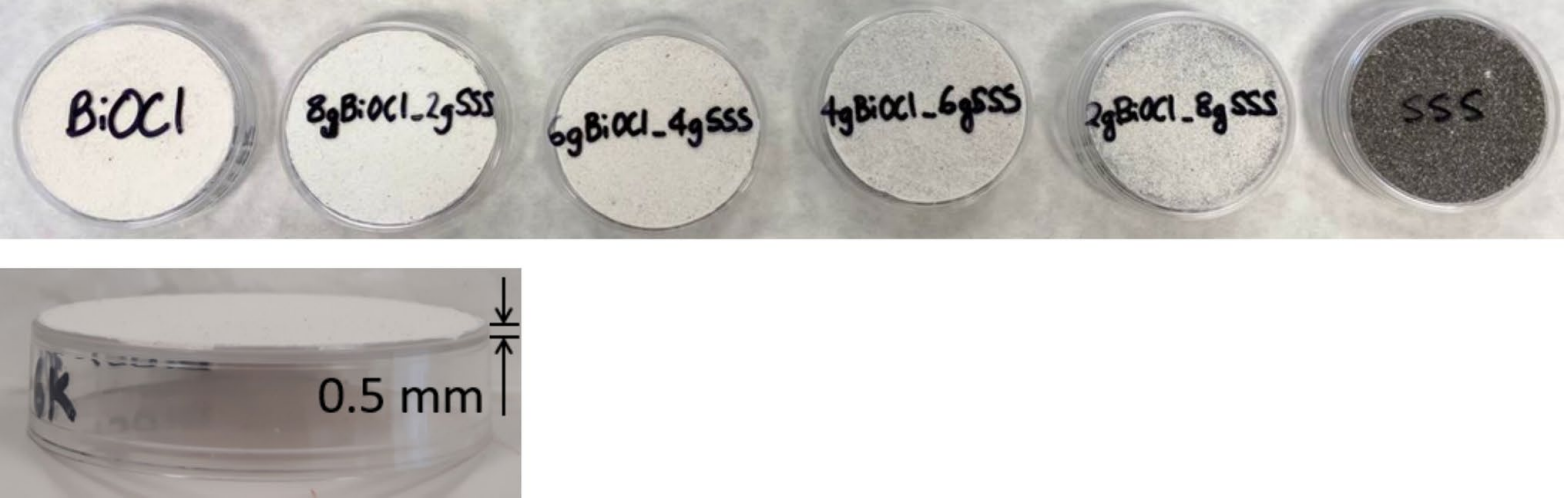
Samples of BiOCl_m_ and BiOCl_m_/SSS composites and SSS for the NOx removal tests

The photocatalytic NOx removal and the selectivity toward nitrate formation (De-NOx selectivity) (Bloh et al. 2014) were evaluated based on an adapted version of the ISO 22197–1 standard. A total of 4 g of each sample was combined with distilled water to form a paste, which was then spread evenly over the bottom of a Petri dish, achieving a layer of 0.5 mm in depth and 5 cm in diameter (Fig. 2). The dry samples were introduced in the continuous flow reactor and tested under two irradiation conditions: UV (Philips TL-D18W BLB) or visible light (Philips master TL-D15W/840), with the spectra shown in Fig. S1(a) and Fig. S1(b), respectively. A continuous laminar flow of NO gas (1000 ± 50 ppb), mixed with air at a flow rate of 3 L·min⁻^1^ and relative humidity of 50 ± 5%, was passed through the reactor at room temperature. Prior to irradiation, the system was kept in the dark for 30 min to allow for adsorption–desorption equilibrium on the sample surface. Photocatalytic activity was then measured over a 30-min illumination period. The concentrations of NO and NO_2_ were continuously monitored using a chemiluminescence analyzer (AC32e).

## Results and discussion

### Thermogravimetric analysis

**Fig. 3:**
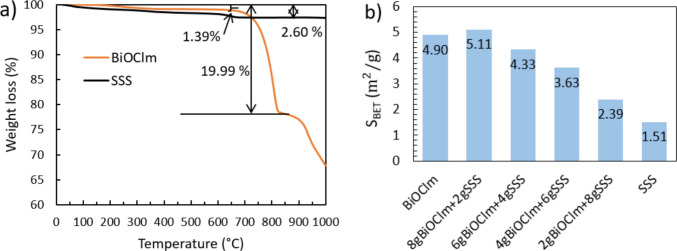
**a** The thermal analysis of BiOCl_m_ and SSS. **b** The Brunauer-Emmett-Teller–specific surface areas (S_BET_) of all studied samples

The thermal stability of BiOCl_m_ and SSS studied in term of weight loss versus temperature is given in Fig. 3a. The weight loss of BiOCl_m_ began only after reaching 200 °C. When the temperature was further increased to 650 °C, 1.39% of weight loss was achieved, which is lesser than the reported solvothermal-synthesized BiOCl showing a weight loss of 5% (A.C. Mera et al. 2020). The weight loss at this temperature could be due to the bismuth oxyhydrochloride [Bi(OH)Cl] decomposition (S. Shamaila et al. 2011) or the change of amorphous BiOCl to crystal BiOCl. When the temperature was further increased from 700 to 800 °C, which involves the decomposition of BiOCl into Bi_2_O_3_, BiOCl_m_ showed a weight loss of 19.99% in comparison with the reported solvothermal-synthesized BiOCl which showed a weight loss of 25% (A.C. Mera et al. 2020), indicating that BiOCl_m_ so synthetized seems to present higher thermal stability. SSS exhibited an immediate weight loss upon heating. However, after reaching 680 °C, the weight loss stabilized at 2.60%, which is primarily attributed to the sequestration of environmental gases (H. Zhang et al. 2021). The enhanced thermal stability of mechanochemically synthesized BiOCl compared to solvothermal synthesized BiOCl may be attributed to several factors inherent to the synthesis method. Mechanochemical synthesis typically leads to materials with fewer residual solvents or organics, and less structural water, which reduces the weight loss at lower temperatures. Additionally, the high-energy ball milling process can induce the formation of more defect-rich or amorphous phases with stronger particle–particle interactions and enhanced crystallite cohesion. These features may delay phase transformations or decomposition processes upon heating. In contrast, solvothermal synthesis often results in more crystalline materials with loosely bound surface species or residual hydroxyl groups, which can volatilize or decompose at lower temperatures, thus contributing to a higher observed weight loss.

### N_2_ adsorption–desorption isotherm

The N_2_ adsorption–desorption isotherms of BiOClm and SSS, shown in Fig. S2(a–f), exhibit a type II behavior. The isotherm of BiOClm shows minimal hysteresis, suggesting a limited presence of mesopores and a predominantly non-porous or microporous surface structure. This contrasts with the slit-shaped or cylindrical pores typically reported for solvothermal-synthesized BiOCl (G. Li et al. 2014; X. Gao et al. 2015; Y. Zhang et al. 2022). In contrast, the isotherm of SSS displays a clear hysteresis loop in the *P*/*P*_0_ range of 0.45 to 1.00, indicating the presence of slit-shaped mesopores on its surface. A small hysteresis loop also begins to emerge and becomes more pronounced in the BiOCl_m_/SSS composites as the amount of SSS increases (Fig. S2(b–e)), attributed to the porous contribution of the SSS component. The inset in Fig. S2(a-f) is the Barrett-Joyner-Halenda (BJH) pore diameter distribution curve for each sample. The majority of the surface pores of BiOCl_m_ are mesopores centered mainly at the pore diameter of 2.2 nm where a maximum sharp peak takes place. BiOCl_m_ nanoplates in this study synthesized mechanochemically tend to shift the maximum of the pore size distribution toward smaller size in comparison with that reported using other synthesis methods (G. Li et al. 2014; Y. Zhang et al. 2022). The BJH pore diameter distribution of SSS, shown as the inset in Fig. S2(f), exhibits a broad curve ranging from 2.6 to 100 nm. This suggests the presence of both mesopores (2–50 nm), centered around 12.2 nm, and macropores (> 50 nm), with the mesopores being the majority and the macropores the minority. As the amount of SSS increased, the peak at 12.2 nm became more prominent and broadened in the BJH pore diameter distribution of the BiOClm/SSS composites. This indicates an increase in macropore content within the composites, attributed to the contribution of SSS. The SSS exhibits slit-shaped pores in its surface structure, some of which have pore diameters greater than 50 nm, which likely facilitate the firm anchoring of BiOCl_m_ onto its surface.

Figure 3b presents the Brunauer–Emmett–Teller–specific surface areas (S_BET_) of the samples. As a general trend, a higher content of SSS in the composite leads to a smaller S_BET_. The S_BET_ of BiOCl_m_ synthetized in this study was 4.90 m^2^/g, which is lower than that of the reported solvothermal-synthesized BiOCl spheres (S_BET_ = 10.29 m^2^/g) (P. Zou et al. 2021) and the hydrothermal-synthesized BiOCl nanoplate (S_BET_ = 6.9 m^2^/g) (J. Guo et al. 2022).

### Scanning electron microscopy

**Fig. 4:**
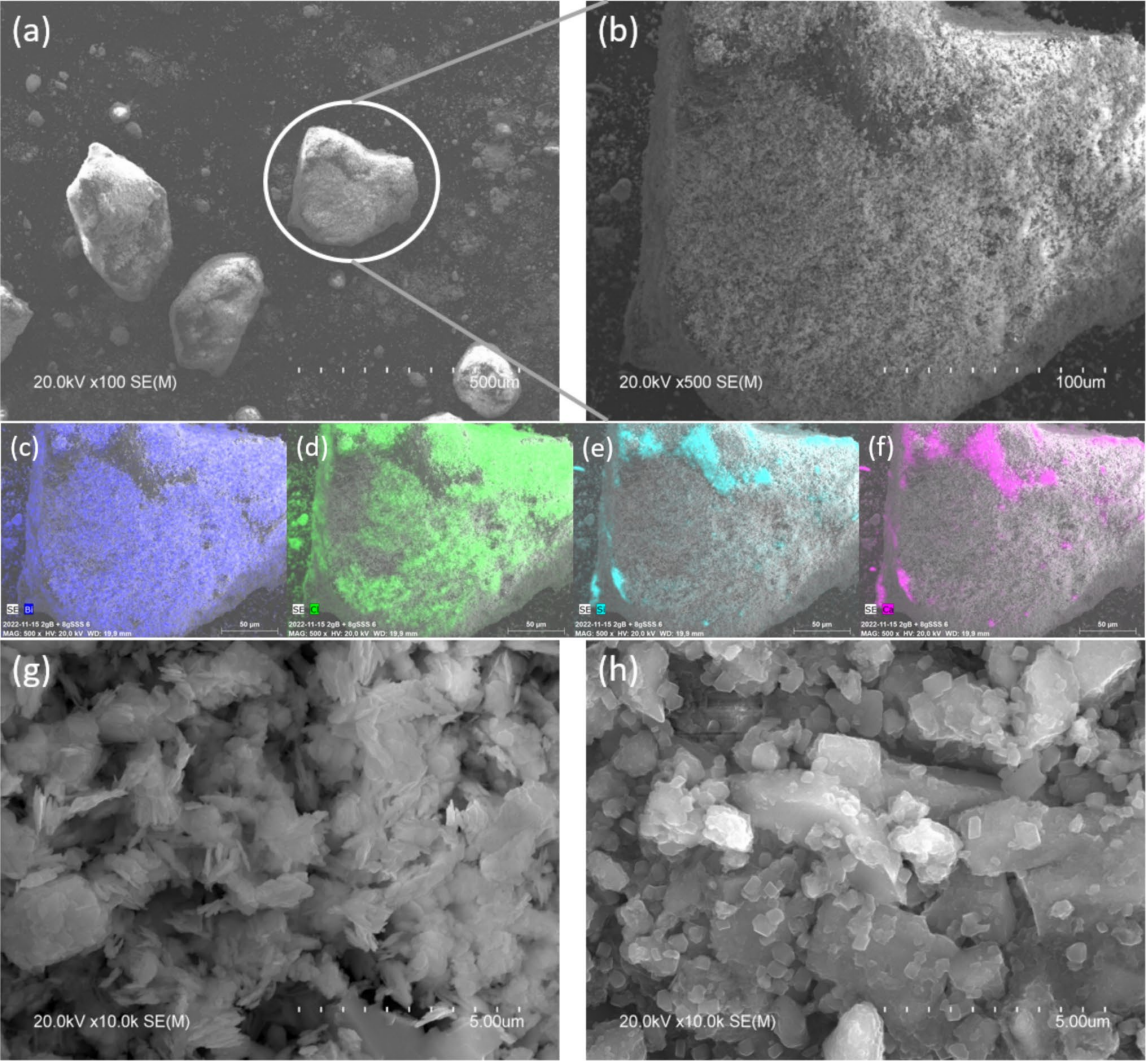
**a** SEM image of SSS grains coated with BiOCl_m_. **b** A × 500 magnification of a grain from **a**. **c**–**f** Elemental mappings of the magnified grain with **c** Bi in purple, **d** Cl in green, **e** Si in blue, and **f** Ca in pink. **g** × 10 k magnification of BiOCl_m_ coated on SSS and **h** × 10 k magnification of SSS fine powder

Figure 4a shows a SEM image of some grains taken from the BiOCl_m_/SSS composites and Fig. 4b presents a magnification at × 500 of a grain from Fig. 4a. The elemental mappings of the grain in Fig. 4b are given in Fig. 4c–f, revealing that the surface of SSS particles is successfully coated by BiOCl_m_. The image of BiOCl_m_ coated on SSS at higher magnification (× 10 k) is shown in Fig. 4g and that of SSS fine powder in Fig. 4h. SSS appeared as rectangular crystallites and BiOCl_m_ appeared as flaky-shaped nanoplates. This confirms the successful deposition of BiOCl_m_ on SSS mechanochemically.

### X-ray diffraction

**Fig. 5:**
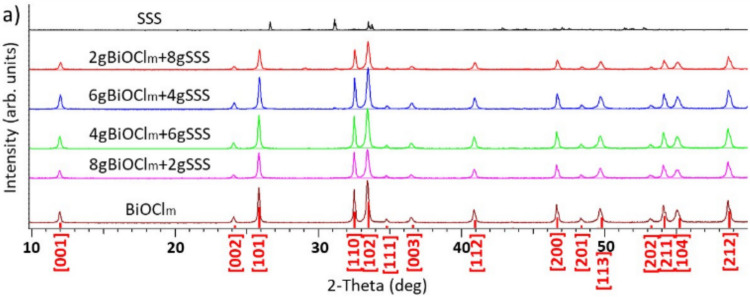
XRD patterns of BiOCl_m_, BiOCl_m_/SSS composites and SSS

The XRD patterns of BiOCl_m_, BiOCl_m_/SSS composites, and SSS are presented in Fig. 5. The specific mineral phases of bare SSS have been described in previous works (Plaza et al. 2021). It can be seen that the diffraction peaks of SSS between 26.5 and 32° appeared only weakly in the 2gBiOCl_m_ + 8gSSS composite.

### Fourier transform infrared spectroscopy (FTIR)

The FTIR spectra of BiOCl_m_, BiOCl_m_/SSS composites and SSS are presented in Fig. S3. A broad absorption peak at 3447 cm^−1^ corresponding to the stretching mode of the hydroxyl (–OH) group was observed in the spectrum of each sample. This reveals that BiOCl_m_ and BiOCl_m_/SSS composites as well as SSS might have a good adsorption ability for water molecules (P. Zou et al. 2021). Thus, this indicates that the absorption of water molecules occurs on the surface of the sample, leading to the formation of the –OH groups that could hook on the surface functional groups of the pollutants during the photocatalytic activity (D.-H. Wang et al. 2012). On the other hand, two adsorption peaks of 1430 and 1640 cm^−1^ observed are due to carboxyl groups (Rahmat et al. 2018; Q. Geng et al. 2012). In the spectrum of SSS, an absorption peak at 512 cm^−1^ and a broad absorption peak at 1022 cm^−1^ could be associated to the bending mode of Al–O–Si and to the antisymmetric in-plane Si–O–Si, respectively (J. Madejová et al. 2017). In the spectrum of BiOCl_m_, a strong and sharp absorption peak at 530 cm^−1^ is associated to the symmetrical vibrations of Bi–O bond (X. Gao et al. 2015). This peak became slightly broader in the BiOCl_m_/SSS composites, especially 2gBiOCl_m_ + 8gSSS, when the amount of SSS increases, which might be due to a mixed vibration of Al–O–Si and Bi–O bond. A weak broad peak at 1039 cm⁻^1^ might be associated with oxygen vacancies in BiOCl (P. Zou et al. 2021). As the amount of SSS increases, the oxygen vacancy peak in BiOCl_m_/SSS composites, especially in 2gBiOCl_m_ + 8gSSS, becomes similar to that appearing in the spectrum of SSS, which could be due to the dominant vibration of Si–O–Si in 2gBiOCl_m_ + 8gSSS.

### Optical properties

**Fig. 6:**
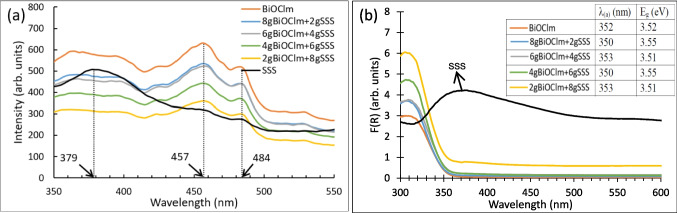
**a** Photoluminescence (PL) spectra and **b** UV-Vis absorption spectra of BiOCl_m_, BiOCl_m_/SSS composites and SSS

Figure 6a demonstrates the photoluminescence (PL) spectra of the samples studied in this work with the excitation wavelength at 315 nm. SSS shows an emission peak at 379 nm that did not appear in the BiOCl_m_/SSS composites, further indicating that BiOCl_m_ is anchored on the surface of SSS grains. BiOCl_m_ and BiOCl_m_/SSS composites showed two emission peaks at 457 and 484 nm that presumably reflect the occurrence of the recombination of photoexcited electrons and holes in the samples. The intensity of these two peaks decreases as the amount of BiOCl_m_ in the sample is reduced.

Figure 6b shows the absorption spectra of all samples after applying the Kubelka–Munk absorption function (F(R_∞_)) on the diffuse reflectance spectra obtained from the UV–Vis spectrophotometer. All the samples showed a good absorption ability in the ultraviolet region. The SSS sample displays very high absorption in the visible range due to its dark color. This absorption progressively decreases as the amount of SSS is reduced and BiOCl is anchored onto the surface of the SSS grains in the BiOCl_m_/SSS composites, eventually leading to no visible-light absorption in the pure BiOCl_m_ sample. The wavelength at the absorption edge (*λ*_(a)_) is displayed in the inserted table in Fig. 6b, together with its corresponding band-gap energy (*E*_*g*_) calculated using the Planck-Einstein relation (Makuła et al. 2018). SSS, as expected, exhibits a shape related with their dark color, which does not allow to define a clear band gap.

**Fig. 7:**
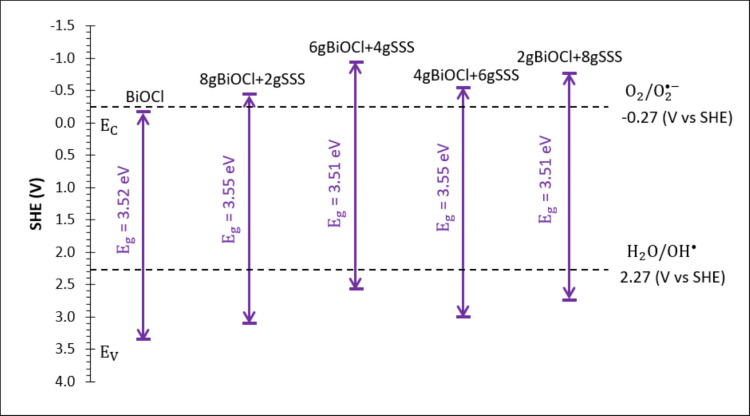
Schematic diagram of band-edge positions of BiOCl_m_ and BiOCl_m_/SSS composites

Figure 7 shows the band-edge positions of all the samples under UV light constructed using the Mott-Schottky analysis (S.F. Lee et al. 2022). Table S1 shows the flat band potential (*V*_fb_) and charge carrier density (*N*_*d*_) obtained from the *x*-axis intercept point and the *n*-type slope of the Mott-Schottky plot. The values of *N*_*d*_ obtained were in the order of 10^19^ and 10^20^. From the band-edge position, BiOCl_m_ could produce hydroxyl radical (OH·) from the water oxidation only at the valence band, but not superoxide radical anion ($O_2^{\cdot-}$) from the dissolved oxygen gas at the conduction band. It can be seen that when BiOCl_m_ was anchored on SSS, the band-edge positions of BiOCl_m_/SSS composites moved upwards, allowing both $O_2^{\cdot-}$ and OH· to be produced simultaneously in the BiOCl_m_/SSS composites. The sample with higher reducing power of the CB is again 6gBiOCl_m_/4gSSS.

The elemental composition of SSS by X-ray fluorescence (XRF) is shown in Fig. S4, where it can be seen that their major component is CaO (≈ 51%) followed by SiO (≈ 26%). Then, there are some elements whose oxides can be semiconductors, that in descending order of quantity, as oxides, are MgO (≈ 8.6%, *E*_*g*_ = 3.45 (Abdullah et al. 2023)), Al_2_O_3_ (≈ 5%, *E*_*g*_ = 2.83–2.90 (A.K. Kaviti and S.R. Akkala 2023), Cr_2_O_3_ (≈ 3.6%, *E*_*g*_ = 3.33–3.50 (Haryński et al. 2022; Xu and Schoonen 2000)) and other minor components below the 2% as MnO (≈ 2%) TiO_2_ (≈ 1.5) and Fe_2_O_3_ (≈ 1.4%), some of the semiconductors are able to adsorb in the visible-light energy range. As some examples, the CB position of MnO was proposed to be highly reductive at around − 1.0 V SHE (Xu and Schoonen 2000) and defective Al_2_O_3_ were proposed at − 0.33 V SHE (Li et al. 2016), able to produce $O_2^{\cdot-}$. In addition, in SSS, there are spinels including different elements that can contribute in some way to the shift of the energy levels of the bands, and also heterojunctions might be formed, which would be in agreement with the lower recombination in the case of the composites. More research is needed to further clarify this aspect.

### Photocatalytic NOx abatement

The concentration profiles of NO gas as a function of time during the experiments are presented in Fig. S5. As observed, the NO concentration remained stable during the dark phase, indicating no reaction between the adsorbed species and the photocatalyst. Upon irradiation with UV or visible light, a rapid decrease in NO concentration was recorded for both BiOClm and BiOClm/SSS composites, with a more pronounced drop under UV irradiation, suggesting enhanced photocatalytic activity in this spectral range.

**Fig. 8:**
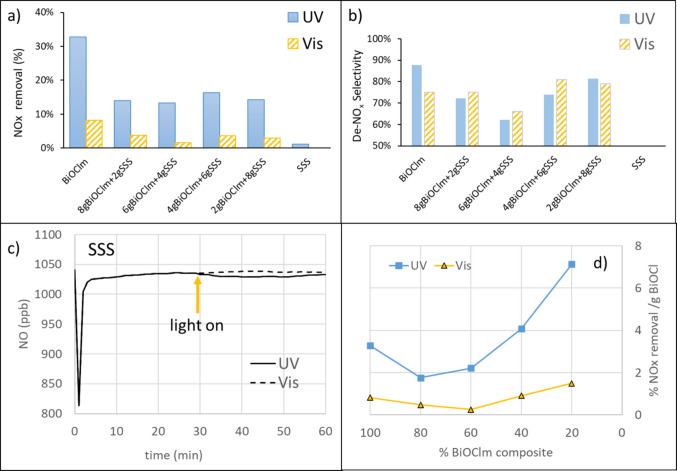
**a** NOx removal (%); **b** De-NO_x_ selectivity of BiOCl_m_, BiOCl_m_/SSS composites, and SSS under UV and visible light. **c** Details of abatement of NO for SSS. **d** NOx removal (%)/g BiOCl

Figure 8a summarizes the NOx removal efficiencies (%) of the different samples under UV and visible irradiation. Pure BiOCl_m_ exhibited higher removal efficiency than the BiOCl_m_/SSS composites under both irradiation conditions. Interestingly, the efficiency of the BiOCl_m_/SSS composites did not appear to follow a systematic trend with increasing BiOCl_m_ content, suggesting that factors beyond simple loading influence photocatalytic behavior. By comparing all the samples, 4gBiOCl_m_ + 6gSSS had the highest NOx removal efficiency under UV while 8gBiOCl_m_ + 6gSSS had the highest NO removal efficiency under visible light. SSS presents a very slight NOx removal under UV light (Fig. 8c). When normalizing the removal efficiency per gram of catalyst (Fig. 8d), samples containing 20% and 40% BiOClm demonstrated higher NOx removal efficiency per gram than pure BiOClm, particularly under UV irradiation, highlighting the beneficial effect of integrating SSS as a support in certain proportions. Regarding the De-NOx selectivity (Fig. 8b), under UV irradiation, BiOClm showed the highest conversion of NO to nitrate (88%), followed by 2gBiOCl_m_ + 8gSSS (81%) and 4gBiOCl_m_ + 6gSSS (74%). However, under visible light, BiOClm exhibited a lower selectivity (75%) compared to the composites. In this case, 4gBiOCl_m_ + 6gSSS reached the highest selectivity (81%) among the composites.

The higher selectivity toward nitrate under UV irradiation can be attributed to the more efficient generation of reactive oxygen species (ROS), such as OH· and/or $O_2^{\cdot-}$, due to the higher energy of UV photons. These ROSs play a crucial role in the complete oxidation of NO to nitrate, minimizing the formation of undesired intermediates like NO_2_. Under visible light, the lower photon energy may lead to less efficient charge separation and reduced production of ROS, especially in pure BiOCl. As a result, the oxidation process may stop at intermediate stages, decreasing the overall selectivity toward nitrate. This effect is partially mitigated in the composites, where the presence of SSS can enhance light absorption and charge separation, improving selectivity under visible light, although still not reaching the efficiency observed under UV.

**Fig. 9:**
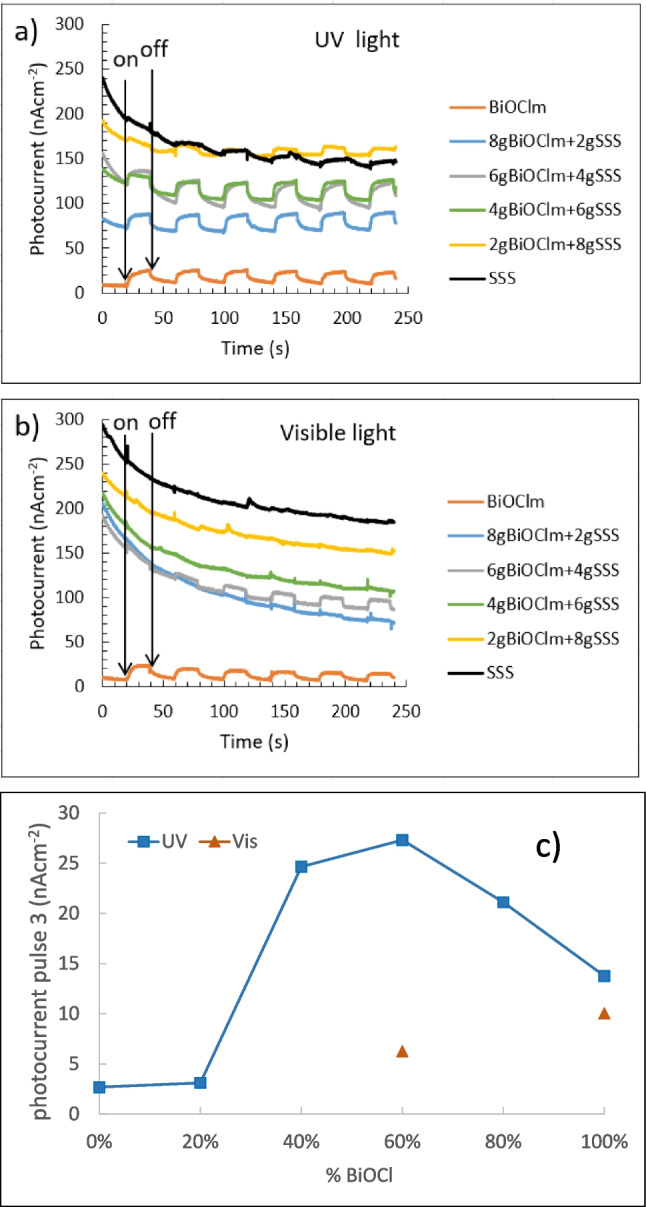
Photocurrent of BiOCl_m_ and BiOCl_m_/SSS composites and SSS under on–off illumination of **a** UV (detail of pulse 3) and **b** visible light. **c** Jump of current when switching on the light (pulse 3)

In order to understand better the results obtained, photocurrent studies have been carried out. Figure 9a and b show the photocurrent-time characteristic curves of BiOCl_m_ and BiOCl_m_/SSS composites and SSS under UV or visible light, respectively. Notice that the baseline in dark is higher than the content of SSS in the sample. Under UV illumination (Fig. 9a), it can be seen that the photocurrent amplitudes remained consistent over the six cycles. The sample of pure SSS did not show the definite pattern that can be found for the samples with BiOCl_m_ composites under on–off UV illumination; however, photocurrent enhanced jumps are detectable and quantifiable. The detail in Fig. 9a presents the jump for cycle 3, whose quantification is given in Fig. 9c, where the increase in current when irradiating, in function of the percentage of BiOCl_m_ in the sample, has been depicted. For UV light, it is remarkable that the photocurrent reached with 60% of BiOCl_m_ is higher than that of pure BiOCl_m_. With visible light, there is a photocurrent generation clearly noticeable for BiOCl_m_ and 6gBiOCl_m_ + 4gSSS, which might be attributed to the transition of photoexcited electrons from the valence band to oxygen vacancies located below the conduction band (G. Liu et al. 2022).

**Fig. 10:**
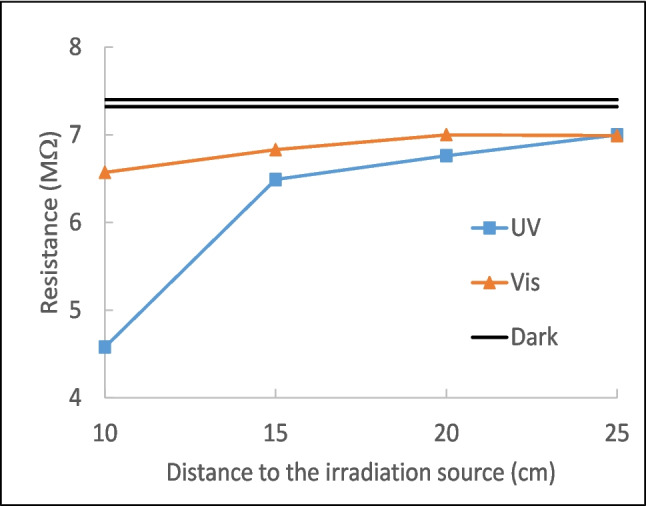
Electrical resistance of SSS under UV and Vis irradiation at different distances from the source

To deepen into the behavior of SSS under irradiation, the electrical resistance of SSS in dark and under irradiation has been measured. To do so, two carbon electrodes were plunged into a petri dish full of SSS, and resistance was measured at different distances from the UV or visible light source. The results are given in Fig. 10, where it can be seen that resistance decreases when increasing the power of the light (~ distance of the irradiation source), in a longer extent with UV light. Thus, it could be said that SSS presents some semiconductor character in agreement with the photocurrent tests that can be enhanced by the photocatalytic performance of BiOCl support on SSS. This could be due to coupling between them that causes the band energy positions to shift upward; BiOCl_m_ could produce OH· from the water oxidation, but not $O_2^{\cdot-}$ from the dissolved oxygen gas at the conduction band. BiOCl_m_/SSS composites can produce both $O_2^{\cdot-}$ and OH· simultaneously, possibly due to the contribution of semiconducting SSS compounds or heterojunction formation. According to the results, a proportion of SSS in the range 40–60% would be the most convenient to be used as active support of BiOCl_m_.

Overall, using waste slag as a support material offers significant economic and environmental advantages compared to conventional substrates. From an economic perspective, waste slag is an abundant and low-cost byproduct of industrial processes, reducing the overall material costs associated with conventional support production. Environmentally, repurposing slag contributes to circular economy strategies by diverting industrial waste from landfills and minimizing the environmental footprint of material fabrication. Moreover, this approach aligns with sustainability goals by promoting the use of recycled resources, lowering raw material consumption, and supporting greener manufacturing practice.

## Conclusions

In the context of the transition towards a circular economy and sustainable development, this work presents the results regarding a BiOCl photocatalyst immobilized on of stainless-steel slag waste (SSS). The photocatalytic functionalization was achieved by depositing various concentrations of mechanochemically synthesized BiOCl (BiOCl_m_) on its surface using an ultrasonication technique. This study revealed that the BiOCl_m_/SSS material behaved as a photocatalyst for NOx air oxidation, showing lower absolute efficiency for both UV and Vis irradiation, but higher efficiency in relation to the weight of BiOCl_m_ in each sample. SSS demonstrated some semiconducting character when activated by UV light, exhibiting enhanced photocurrent signals and minimal NOx degradation (around 1%) on its own. Thus, the coupling or heterojunction formation between BiOClm and SSS causes the band energy positions to shift upward. According to the results, an SSS proportion of 20–40% would be the most suitable for use as an active support for BiOClm. These findings demonstrate that replacing conventional supports with waste slag offers a cost-effective and sustainable alternative by repurposing industrial waste and reducing environmental impact. The integration of industrial byproducts into advanced photocatalytic materials reinforces the potential of slag-based systems as a promising, scalable, and eco-efficient solution for environmental remediation applications.

## Supplementary Information

Below is the link to the electronic supplementary material.

ESM 1 (DOCX 496 KB)

## Data Availability

No data was used for the research described in the article.
