# Supplementary material for: Bismuth oxychloride anchored on waste stainless-steel slag: preliminary assessment of its potential as an active photocatalytic support
Source: Environ Sci Pollut Res Int. 2025 Jun 24;32(27):16249–60. doi: 10.1007/s11356-025-36656-1 (PMC12274146; doi:10.1007/s11356-025-36656-1)
Supplement: Supplementary file 1 — (DOCX 496 KB) [file 11356_2025_36656_MOESM1_ESM.docx]

**Supplementary Information**


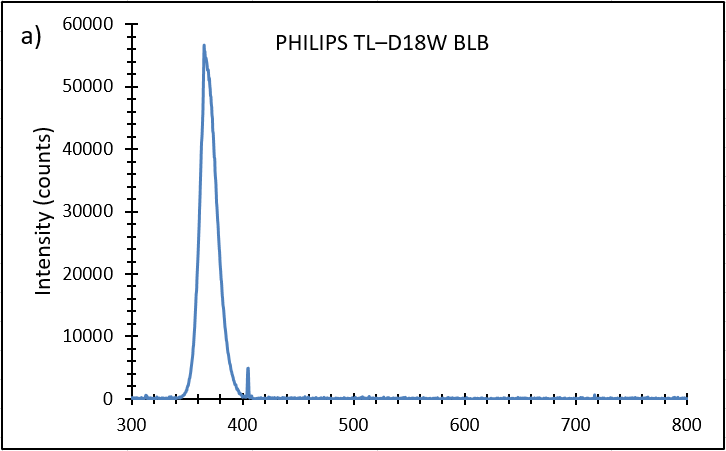

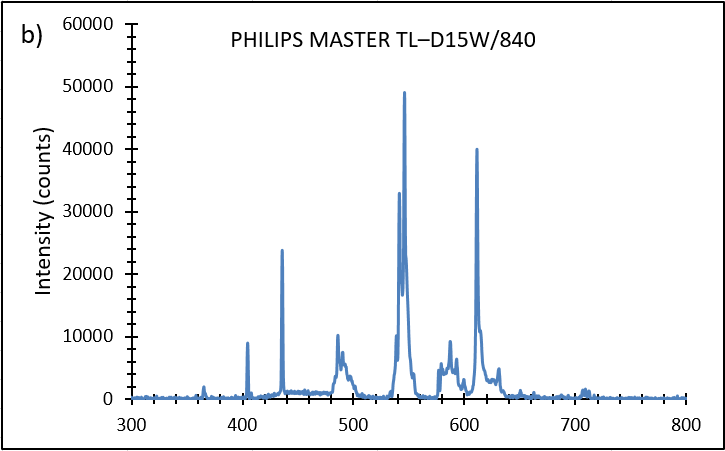


**Fig. S1** Spectrum of (a) PHILIPS TL-D18W BLB, and (b) PHILIPS MASTER TL-D15W/840.


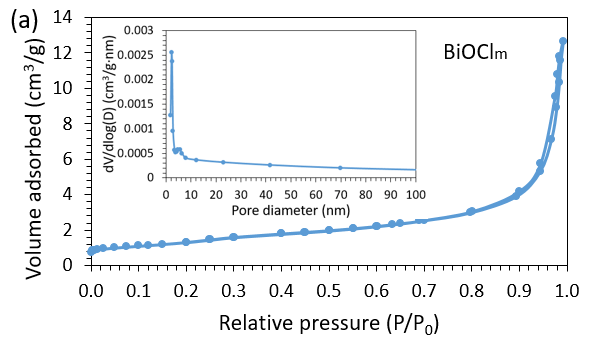

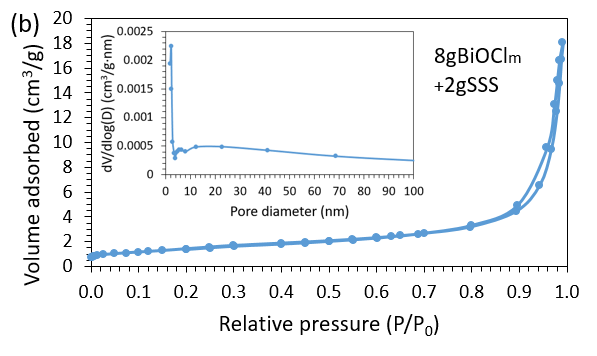


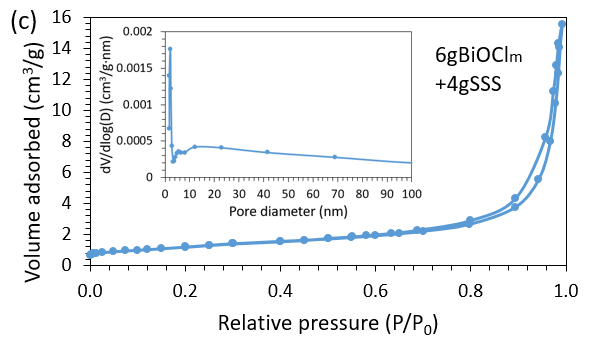

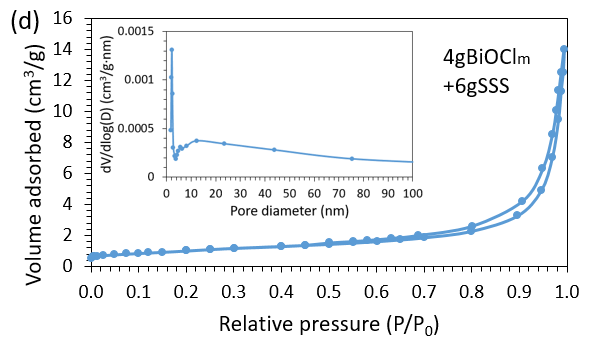

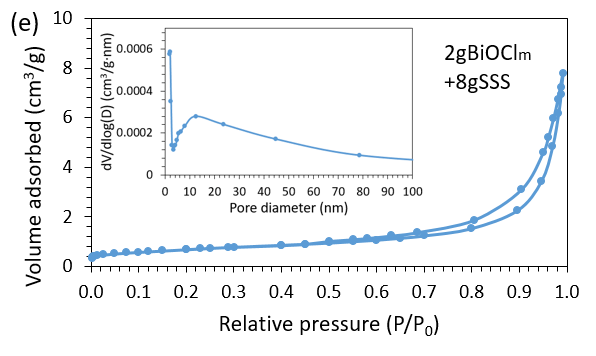

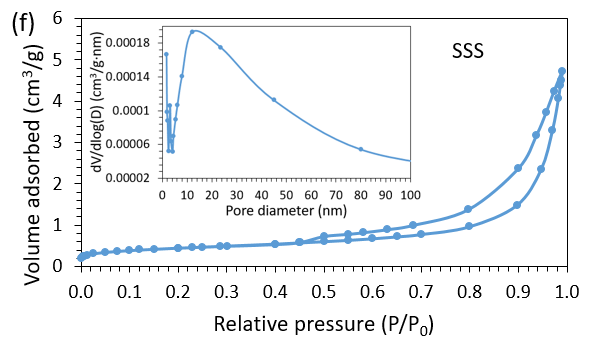
**Fig. S2** N_2_ adsorption-desorption isotherms and the corresponding BJH pore diameter distribution curves an inset of (a) BiOCl_m_, (b) 8gBiOCl_m_+2gSSS, (c) 6gBiOCl_m_+4gSSS, (d) 4gBiOCl_m_+6gSSS, (e) 2gBiOCl_m_+8gSSS, and (f) SSS.


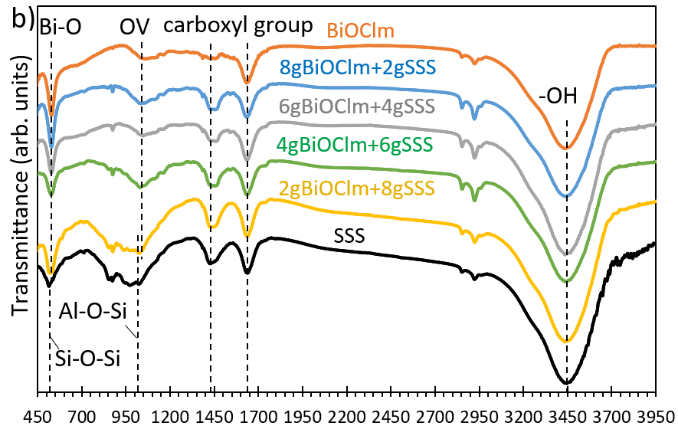


**Fig. S3** FTIR spectra of BiOCl_m_, BiOCl_m_/SSS composites and SSS.

**
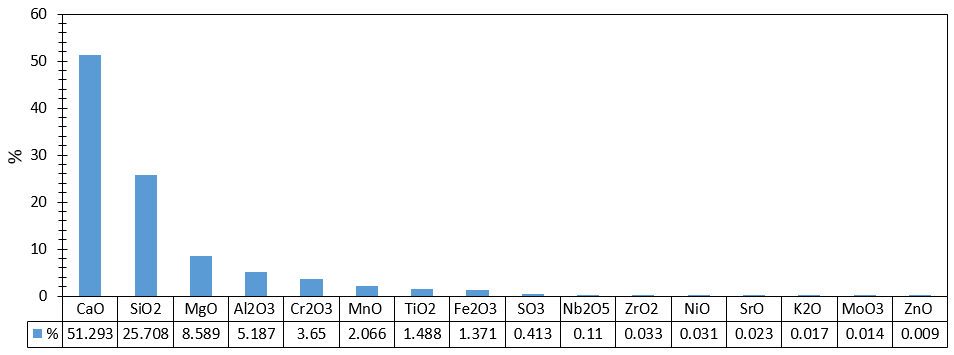
**

**Fig. S4** Elemental-composition analysis of raw SSS


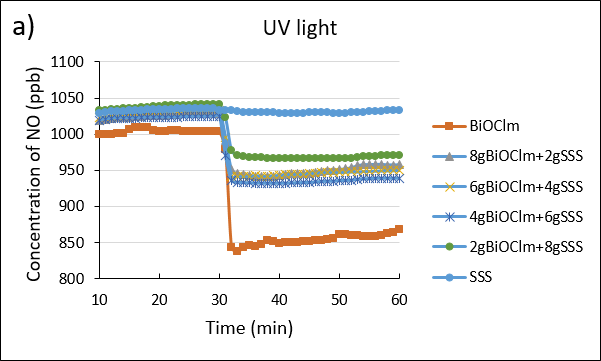

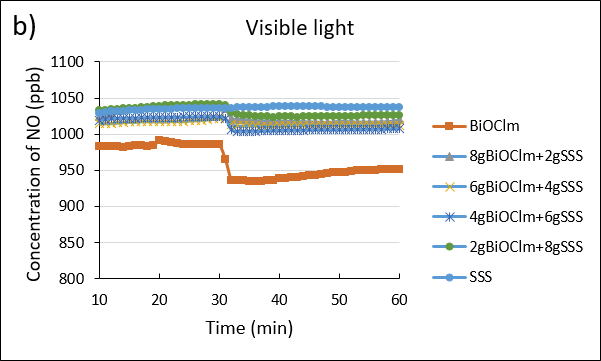


**Fig. S5** NO concentration evolution of BiOCl_m_, BiOCl_m_/SSS composites and SSS in the dark for 30 min and after the UV or visible light is on.

**Table S1.** Flat-band potential (V_fb_), conduction band (E_C_), band-gap energy (E_g_), valence band (E_V_), donor charge concentration (N_d_) of BiOCl_m_, BiOCl_m_/SSS composites and SSS under UV. E_C_ is obtained by converting V_fb_ to the SHE scale and E_V_ is calculated from E_C_ using E_g_.

| UV light | Mixture | f  (Hz) | V_fb_  (V vs Ag/AgCl) | E_C_  (V vs SHE) | E_g_  (eV) | E_V_  (V vs SHE) | N_d_  (cm^-3^) |
| --- | --- | --- | --- | --- | --- | --- | --- |
|  | BiOCl | 1000 | -0.37 | -0.165 | 3.52 | 3.355 | 6.5620 x 10^19^ |
|  | 8gBiOCl+2gSSS | 1000 | -0.65 | -0.445 | 3.55 | 3.105 | 1.0784 x 10^20^ |
|  | 6gBiOCl+4gSSS | 1000 | -1.16 | -0.955 | 3.51 | 2.555 | 1.5950 x 10^20^ |
|  | 4gBiOCl+6gSSS | 1000 | -0.75 | -0.545 | 3.55 | 3.005 | 9.7880 x 10^19^ |
|  | 2gBiOCl+8gSSS | 1000 | -0.97 | -0.765 | 3.51 | 2.745 | 1.3410 x 10^20^ |
|  | SSS | 1000 | -1.14 | -0.935 | 4.57 | 3.635 | 1.8640 x 10^20^ |
